# How Far Is the Nanocellulose Chip and Its Production in Reach? A Literature Survey

**DOI:** 10.3390/nano14181536

**Published:** 2024-09-22

**Authors:** Elena Bencurova, André Chinazzo, Bipasa Kar, Matthias Jung, Thomas Dandekar

**Affiliations:** 1 Functional Genomics and Systems Biology Group, Department of Bioinformatics, Biocenter, Am Hubland, University of Würzburg, 97074 Würzburg, Germany; elena.bencurova@uni-wuerzburg.de (E.B.); bipasa.kar@uni-wuerzburg.de (B.K.); 2 Microelectronic Systems Design Research Group, Department of Electrical and Computer Engineering, University of Kaiserslautern-Landau, 67663 Kaiserslautern, Germany; chinazzo@rptu.de; 3 Computer Engineering, Institute for Computer Science, University of Würzburg, 97074 Würzburg, Germany; 4 Embedded Systems Engineering, Fraunhofer IESE, 67663 Kaiserslautern, Germany; 5 European Molecular Biology Laboratory, Structural and Computational Biology Unit, 69117 Heidelberg, Germany

**Keywords:** computer engineering, nanocellulose, EDA, organic-based transistors

## Abstract

The slowdown of Moore’s Law necessitates an exploration of novel computing methodologies, new materials, and advantages in chip design. Thus, carbon-based materials have promise for more energy-efficient computing systems in the future. Moreover, sustainability emerges as a new concern for the semiconductor industry. The production and recycling processes associated with current chips present huge environmental challenges. Electronic waste is a major problem, and sustainable solutions in computing must be found. In this review, we examine an alternative chip design based on nanocellulose, which also features semiconductor properties and transistors. Our review highlights that nanocellulose (NC) is a versatile material and a high-potential composite, as it can be fabricated to gain suitable electronic and semiconducting properties. NC provides ideal support for ink-printed transistors and electronics, including green paper electronics. Here, we summarise various processing procedures for nanocellulose and describe the structure of exclusively nanocellulose-based transistors. Furthermore, we survey the recent scientific efforts in organic chip design and show how fully automated production of such a full NC chip could be achieved, including a Process Design Kit (PDK), expected variation models, and a standard cell library at the logic-gate level, where multiple transistors are connected to perform basic logic operations—for instance, the NOT-AND (NAND) gate. Taking all these attractive nanocellulose features into account, we envision how chips based on nanocellulose can be fabricated using Electronic Design Automation (EDA) tool chains.

## 1. Introduction

Cellulose, the most abundant natural polymer on Earth, is a linear biopolymer composed of anhydroglucose units with varying degrees of polymerization. Depending on its source and processing method, cellulose can consist of several thousand glucose units. The extensive intra- and inter-molecular hydrogen bonds within cellulose enable it to form strong microfibrils [1,2], making it suitable for applications in the food industry, cosmetics, medicine, and technical fields [3,4]. Moreover, cellulose’s renewability, biocompatibility, and non-toxicity offer distinct advantages over synthetic materials commonly used in electronic devices [5]. Materials derived from cellulose fibres can exhibit different physical properties, despite sharing the same fundamental chemical structure [2]. Although cellulose is a linear, unbranched polymer, it can assume various structural configurations at the molecular level, resulting in multiple allomorphs. The interactions within the crystalline and amorphous domains, driven by various factors, including hydrogen bonding and the degree of polymerization, impart distinct mechanical properties to the allomorphs. For instance, a higher degree of crystallinity generally correlates with increased tensile strength and thermal stability [6].

Nanocellulose (NC) consists of nano-sized cellulose fibrils and can be obtained from various cellulosic sources, including plants and algae, and be produced by bacterial species such as *Agrobacterium* [7], *Gluconacetobacter* [8], and *Rhodococcus* [9]. Bacterial nanocellulose (BNC) is secreted extracellularly, forming a floating pellicle in static cultures that aids immobilisation on the surface, facilitating aeration, and protects against dehydration and microbial competition [10,11]. The quality of BNC depends on the cultivation method; static cultures produce high-quality cellulose but pose scalability challenges, whereas agitated fermentation is more scalable but often yields cellulose with inferior properties [12]. BNC offers significant advantages over plant-derived cellulose, including higher purity, crystallinity, and polymerization [13,14]. On the other hand, BNC is less thermally stable [15], although its properties can be enhanced through chemical or polymer treatments. Its production as a hydrogel is advantageous for applications requiring a never-dried state; however, its drying can lead to irreversible agglomeration. Due to its high hydrophilicity, BNC can absorb up to 100 times its weight in water, forming hydrogels with notable thixotropic properties, making it valuable as a thickening agent and suspension stabilizer [16,17,18]. On the other hand, this feature can also be a disadvantage for some of applications, as we discuss later on. Furthermore, BNC’s nanostructure supports interactions with other polymers, nanoparticles, and small molecules, enhancing its utility in biochemistry and material science [19,20,21]. While its hydrogel properties are well-explored, BNC’s potential extends to aerogel synthesis. Nanocellulose aerogels have been used in high-efficiency air filters, cooling systems, and advanced membrane distillation processes, demonstrating superior performance compared to conventional materials [22,23,24].

In recent years, both cellulose and nanocellulose have garnered significant attention in the field of electronics [25,26,27]. A recent study demonstrated versatile uses of organic field-effect transistors (OFETs) using either organic polymers, such as poly(methyl methacrylate) [28], and a copolymer, such as 1,3,5-trimethyl-1,3,5-trivinyl cyclotrisiloxane polymerized with 1-vinylimidazole [29], or even cellulose thin film or a semiconducting layer coated onto a cellulose dielectric [30]. The increasing demand for energy-efficient portable electronic devices such as mobile phones and computers has prompted many studies to pursue low power consumption, high carrier mobility, and reduced off-state current in unipolar n-type field-effect transistors (FETs).

While OFETs present promising solutions [31], they have limitations, such as low intrinsic carrier mobility and loosely pinned off-state current, resulting in a poor current on/off ratio compared to inorganic FETs. These shortcomings lead to a reduced noise margin, higher power consumption in standby mode, and lower processing power. In contrast to this, inorganic n-type FETs exhibit high carrier mobility, a high current on/off ratio, and well-defined saturation characteristics [32]. NC emerges as a versatile material that is easily produced and integrated as a key component of a nanocellulose chip. Its unique electronic properties, whether in isolation or combined with conductive agents, enable it to function as a conductor, insulator, or semiconductor. Furthermore, NC serves as an optimal composite material and support for green paper electronics [33,34,35,36]. For instance, ink-printed electronics such as transistors and other electronic components can be advantageously printed on NC supports.

In this survey, we collected important evidence and published results on p doping and n doping of NC, as well as the modification of NC conductivity, exploring how these properties allow for and facilitate the design of various NC transistor types, such as field-effect and single-electron transistors. A transistor entirely made from NC would not only be environmentally friendly but would also offer significant potential for miniaturisation comparable to that of conventional electronics.

We examine how these attractive properties could enable the fabrication of NC-based chips using Electronic Design Automation (EDA) tool chains. Additionally, we highlight the unique composite-hosting properties of NC for future research, including light-activated enzymes, DNA wires, and DNA storage, concluding that NC holds immense potential as the foundation for a new generation of computer chips.

The contributions of this survey are summarized as follows:

- We survey how the electrical properties of nanocellulose can be influenced either by a conductive agent or n or p doping.
- We present a new concept of how a transistor consisting only of nanocellulose compounds can be created.
- We review the high potential for miniaturisation of nanocellulose from transistors down to single-electron transistors.
- We envision a PDK and EDA design flow for fully biodegradable nanocellulose chips.

The remained of this paper is structured as follows. First, we present the related work and our envisioned nanocellulose transistor in Section 2 (a concept of the nanocellulose transistor, not a demonstrator). In Section 3 we survey the literature on conductive agents and NC. The envisioned PDK and EDA design flow are presented in Section 4. Section 5 reviews further extensions of the NC composite, whereas Section 6 discusses the development steps for the presented NC transistor, leading to the conclusions presented in Section 7.

## 2. Related Work: Nanocellulose Has a High Potential for Microelectronics

Table 1 summarizes the recent achievements by researchers that pave the way to creating organic and sustainable microelectronic devices. We classify these achievements based on the final properties of the fabricated devices. Our focus on nanocellulose-derived materials is based on their wide range of properties and their biodegradability.

A decade ago, NC was investigated as an appropriate material for simultaneous use as a substrate and gate dielectric for flexible thin-film transistors (TFTs) [37]. Noncrystalline cellulose films were derived from cotton wool and formed by the following two methods: (1) solvent casting and evaporation and (2) sheared casting. Both methods resulted in good mechanical stability of the 20 μm NC films. TFTs were built using a relatively costly sequence of radio frequency magnetron sputtering and e-beam deposition steps on both sides of the NC films for the active semiconductor and electrode layers. The films formed via evaporation led to better electrical properties of the transistors due to lower surface roughness and, hence, a better interface between the dielectric and semiconductor materials.

**Table 1:** Summary of recent achievements with respect to sustainable microelectronic devices, with a special focus on applications of nanocellulose-based materials.

Recent Achievements in Sustainable Microelectronics					
Year	Reference	Organic	Biodegradable	Flexible	Nanocellulose
2024	[38]	Yes	Yes	Yes	Substrate
	[39]	Hybrid	Yes	Yes	
	[40]	Hybrid		Yes	
2023	[41]	Yes	Yes	Yes	Substrate and dielectric
	[42]	Yes	Yes	Yes	Dielectric
	[43]	Yes	Yes	Yes	Dielectric
2022	[44]	Yes	Yes	Yes	Substrate and dielectric
	[45]	Yes	Yes	Yes	P-type semiconductor
	[33]	Yes	Yes	Yes	Substrate
	[46]	Yes	Yes	Yes	N- and P-type semiconductor
	[47]	Yes	Yes	Yes	Substrate
	[48]	Hybrid	Yes	Yes	
2021	[49]	Yes	Yes	Yes	Dielectric
	[50]	Yes	Yes		N-type semiconductor
2020	[51]	Hybrid			
2018	[52]	Hybrid	Yes	Yes	Dielectric
2016	[53]	Hybrid	Yes	Yes	Substrate
2014	[37]	Hybrid		Yes	Substrate and dielectric

The authors of [53] printed transistors on NC substrates using ink-jet technology, emphasizing the material’s adaptability and compatibility with commonly used and inexpensive manufacturing processes. It was concluded that NC performs as well as plastic as an inert substrate. Mechanically stable, ultra-thin 2 μm NC substrate films were fabricated in [38].

Ionic Conductive Cellulose Nanopapers (ICCNs) have demonstrated excellent performance in the formation of the dielectric layer between the transistor’s gate and semiconductor layers [52]. ICCN is a nanocellulose-based material specifically designed to function as solid dielectrics in low-voltage organic transistors. With a thickness of about 40 μm, it can operate at gate-source voltages below 2 V, keeping its self-supporting properties such as flexibility and transparency. ICCNs exhibit a high dielectric constant and possess outstanding mechanical properties, including high-temperature resistance and the formation of an ultra-smooth surface. The material proposed by Dai et al. was created from TEMPO-oxidized cellulose derived from softwood pulp, which was pre-treated with trichloro(1H,1H,2H,2H-tridecafluoro-n-octyl)silane and spin-coated with ionic liquids (octadecyltrichlorosilane toluene solution). This material represents an important step towards flexible and green electronics [52].

More recently, both n-type [46,50] and p-type [45,46] semiconductor materials were achieved by the transformation of NC. The authors of [46] showed that it is possible to tune the electrical properties of NC-derived material by controlling the pyrolysis temperature. At a lower temperature of 650 °C, a mostly n-type semiconductor was formed, while higher temperatures of up to 1100 °C resulted in p-type composites. An n-type semiconductor is characterized by an excess of free electrons in its molecular lattice. Conversely, a p-type semiconductor has an excess of holes (lack of electrons) in its lattice. By controlling the pyrolysis temperature of NC, one can control the density of electron-donating furan-like ether structures (greater at lower temperatures—hence, n-type) and electron-withdrawing carbonyl group structures (greater at higher temperatures—hence, p-type) [46].

The utilisation of these materials to build transistors has yet to be explored, but they provide good evidence that a fully complementary technology (p-type and n-type transistors) based on NC is achievable. Complementary technology is associated with high levels of operational reliability and low static power consumption. Furthermore, deviating from complementary technologies may render the current state-of-the-art EDA solutions unusable. Nanocellulose, when combined with other materials like graphene, provides highly desirable properties for flexible electronics [27]. Therefore, nanocellulose-derived materials can form each of the necessary layers (substrate, dielectric, conductor, and semiconductors) in the construction of transistors and chips.

Transistors are used in microelectronic devices as voltage-controlled switches. The voltage at a given point in the circuit is interpreted as a logic (binary) value, i.e., 1 or 0. The most basic logic function is inversion (NOT), where an input logic value of 0 results in an output logic value of 1 and vice-versa. An electronic inverter is built by connecting at least two transistors and is commonly the first test vehicle to benchmark a novel fabrication technology. More complex logic gates, for example, AND and OR gates, require more transistors. They also require a more intricate analysis for their performance evaluation but are necessary for the creation of a *standard cell library* that enables the design of complete digital systems.

In Table 2, we summarise the current research effort with respect to the creation of standard cell libraries for organic transistors. We observe that the fabrication of Organic Field-Effect Transistors (OFETs) is an extremely active area of research (Table 2 includes only a small fraction of recent publications), while research on the next levels of abstraction for electronic design automation (EDA, see Section 4) is increasingly scarce. Two complete open-source projects including process design kits (PDKs) and a minimal standard cell library (including cell characterization) were found [40,51]. However, both of them rely on transistors that are not fully organic instead making use of metals and other unspecified materials (see Table 1). Furthermore, both works include only p-type transistors, which result in more complex logic gate designs (e.g., a p-type-only inverter requires four transistors instead of only two). Other researchers have managed to fabricate n-type OFETs. A technology capable of fabricating both p- and n-type OFETs is known as complementary technology. Rafiee et al. [39] demonstrated such a complementary technology based on waxed paper sheets but did not develop a PDK or a cell library.

In summary, the following two significant gaps remain in the quest for a biodegradable chip design process:

1. A transistor fully based on nanocellulose-derived materials has yet to be fabricated, and
2. A PDK and standard cell library based on fully biodegradable devices have yet to be developed.

Therefore, we propose that transistors entirely based on nanocellulose-derived materials represent a viable alternative for the manufacturing of environmentally friendly chips.

**Figure 1:**
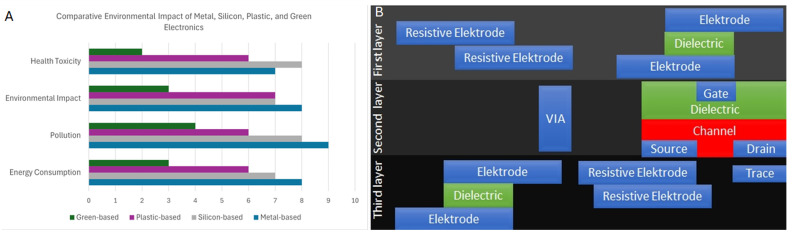
Panel (**A**). Comparison of environmental impacts of various sources of electronic devices based on literature review and data synthesis. The categories on the x axis represent the impact areas, and the y axis shows the normalised impact levels on a scale from 1 to 10. Panel (**B**). Schematic overview of multi-layered paper-tronic circuits. Adapted from Rafiee et al. [39].

Figure 1 shows the most promising route and expected impact of nanocellulose-based circuits. Figure 1A compares the environmental friendliness of green-based, plastic-based, silicon-based, and metal-based electronics. A promising multi-layered approach for the structure of paper/nanocellulose film-based integrated circuits is presented in Figure 1 [39].

**Figure 2:**
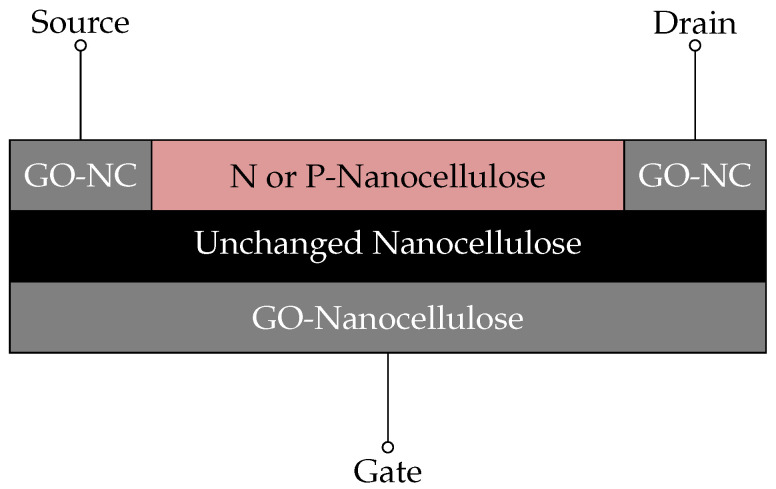
Schematic overview of the proposed transistor using non-treated nanocellulose as a dielectric layer, P- or N-type nanocellulose for the semiconductive channel [45,50], and GO-treated nanocellulose for the conducting parts.

Figure 2 shows the cross-section of the proposed nanocellulose-based transistor (only a sketch, not experimentally tested). By leveraging the broad range of material properties of nanocellulose-derived materials, we claim that both p- and n-type OFETs can be fabricated from an untreated NC substrate based on the results of [45,50]. The electrodes (gate, source, and drain) are composed of NC that is highly doped with graphene oxide (see Section 3), resulting in low-resistance contacts. Either pure nanocellulose or ICCN functions as the dielectric layer between the gate electrode and the organic semiconductor. In the following, we show how nanocellulose can be transformed into a conductor using graphene oxide, among other methods.

## 3. Survey on Nanocellulose Conductivity

Previous research has demonstrated the suitability of NC for various advanced applications, highlighting its potential in the field of semiconductor technologies. The semiconducting properties of iodine-doped NC showcase its potential in electronic devices [21]. Currently, graphene is the most commonly used material to enhance the conductivity of NC and other organic materials. This composite material offers several advantages, and its properties can be influenced by various fabrication methods. Highly conductive materials can be obtained using the one-pot approach [57], with a conductivity of 116.3±1.5 S m^−1^ (at 20 percentage of graphene oxide loading), keeping both thermal stability up to 319 °C and mechanical strength, with a specific tensile strength of 19 N mg^−1^. One notable development is a low-oxidized graphene/nanocellulose hybrid (LGENC), which consists of co-exfoliating graphene and microfibrillated cellulose. This hybrid material exhibits high strength while ensuring elongation, making it a promising candidate for flexible electronics. The interaction between graphene and NC in LGENC is primarily physical, providing an alternative to the commonly used TEMPO-oxidation method for the preparation of NC. Such physical interaction promotes the formation of a robust composite material with excellent mechanical properties [58]. On the other hand, TEMPO oxidation remains a standard approach for producing conductive graphene/cellulose hybrids. TEMPO oxidation introduces carboxylate groups (COO-) onto the cellulose surface, which create electrostatic repulsion and enhance the dispersion of cellulose and NC fibrils or crystals in solution. Additionally, these COO- groups can form covalent bonds with graphene oxide, improving the stability and conductivity of the material [59,60]. By utilizing these advanced fabrication techniques, nanocellulose-based composites can achieve the desired electrical properties for various applications in semiconductor technology and flexible electronics. Additionally, the potential for Single-Electron Transistor (SET) properties in NC was suggested in [21], indicating the material’s ability to support cutting-edge electronic components by further scaling down the technology. These studies collectively underscore the versatility and promise of NC in semiconductor applications and in advancing the development of environmentally friendly and efficient electronic devices. Furthermore, several other materials can be used to enhance the conductive properties of NC. Our previous work [21] identified iodine-doped NC as a conductive material with desired properties for use in electronics. However, the environmental toxicity of iodine, particularly its harmful effects on aquatic organisms, together with its potential reactivity with other materials and degradation over time, make iodine an unsuitable doping agent for semiconductive materials. This shift not only addresses the environmental concerns associated with iodine but also leverages the superior properties of graphene oxide to enhance the performance and sustainability of nanocellulose-based applications.

**Figure 3:**
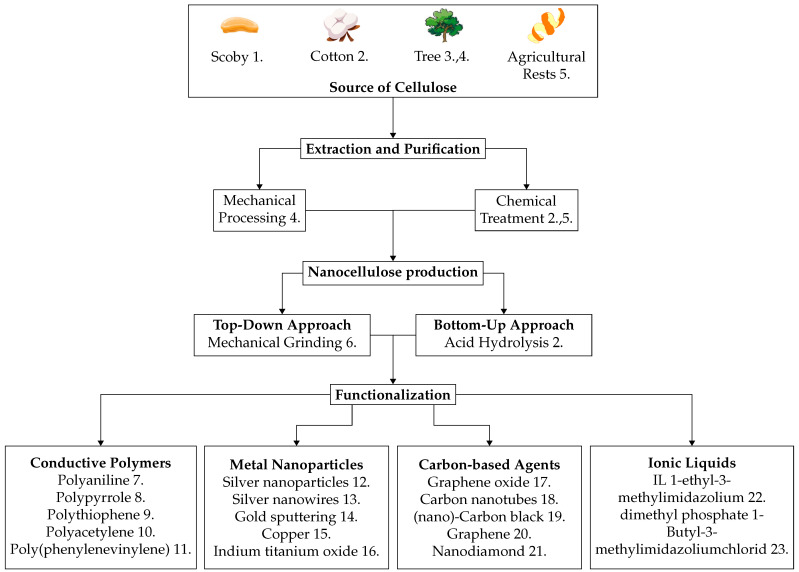
Production and functionalization of nanocellulose for enhanced conductivity. The flow chart illustrates the production and functionalization of NC from biological sources, including Scoby, cotton, trees, and agricultural residues. Extraction and purification can be performed through mechanical processing or chemical treatment, followed by a top-down approach (mechanical grinding) or a bottom-up approach (acid hydrolysis). Functionalization can be achieved by adding conductive materials like conductive polymers, metal nanoparticles, carbon-based agents, and ionic liquids to enhance the electrical properties of nanocellulose for advanced applications. References: 1: [21], 2: [68], 3: [59], 4: [61], 5: [69], 6: [70], 7: [64], 8: [65], 9: [71], 9: [72], 11: [73], 12: [66], 13: [74], 14: [75], 15: [76], 16: [77], 17: [58], 18: [78], 19: [79], 20: [80], 21: [81], 22: [82], 23: [83].

Figure 3 shows the most common methods for manufacturing and processing NC as reported in the literature for use in electronic applications. NC can be prepared using various methods, each offering distinct benefits and drawbacks. Mechanical processing typically involves high-pressure homogenization or ultrasonication to physically break down cellulose fibres into nanoscale dimensions. This method is advantageous due to its simplicity and scalability; however, it typically requires high-energy input and can result in uneven fibre sizes [61]. In contrast, chemical methods, such as acid hydrolysis, treat cellulose with strong acids like sulfuric, hydrochloric, and phosphoric acid to hydrolyze the amorphous regions of cellulose, leaving behind crystalline NC [62]. This approach yields highly crystalline NC with a uniform size and shape, but it raises environmental concerns due to the use of harsh chemicals and the need for neutralization and disposal of acidic by-products. Another chemical treatment, enzymatic hydrolysis, utilizes specific enzymes such as cellulases to selectively break down cellulose fibres into NC [63]. Although enzymatic hydrolysis is more environmentally friendly and energy-efficient than mechanical or chemical methods, it is significantly slower and more costly due to the need for specialized enzymes.

Currently, several materials are being explored to enhance the electrical properties of NC, leading to significant advancements in various fields. Beyond graphene oxide, conductive polymers such as polyaniline (PANI) and polypyrrole (PPy) have shown considerable promise. These polymers can substantially improve electrical performance and can be used to coat cellulose, making them valuable for applications in both electronics and biological studies [64,65]. PANI is particularly known for its tunable conductivity through doping and its high environmental stability. When combined with nanocellulose, PANI enhances the mechanical properties and conductivity of the material, making it an ideal candidate for applications in flexible electronics, sensors, and supercapacitors. Polypyrrole (PPy), on the other hand, exhibits excellent conductivity, ease of synthesis, and biocompatibility. Coating nanocellulose with PPy results in a composite that offers both improved electrical conductivity and chemical stability, making it suitable for applications in energy storage devices and biosensors. Among metals, silver nanoparticles are the most commonly used to boost the electrical properties of cellulose-based composites. They are effective both as standalone additives [66] and when combined with other polymers [67]. When integrated with nanocellulose-based materials, silver nanoparticles form highly conductive networks, significantly improving overall conductivity. In addition to enhanced antimicrobial activity, silver nanoparticles help improve composites’ thermal stability.

Metal oxides like zinc oxide (ZnO) [84] and titanium dioxide (TiO_2_) [85] have also been explored for their potential to enhance these properties. ZnO has highly semi-conductive properties. TiO_2_, with a high refractive index and photocatalytic properties, improves the electrical performance and durability of nanocellulose composites. Thus, all these materials not only improve the electrical characteristics of NC but also contribute to its structural integrity and functionality in diverse applications. These advancements underscore the versatility and potential of conductive NC, positioning it as a key material for future innovations in flexible electronics, sensors, and other high-performance applications.

**Figure 4:**
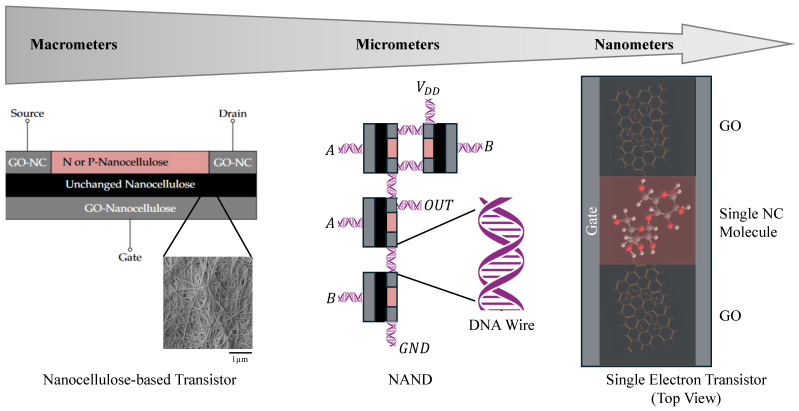
Schematic representation of nanocellulose-based transistor designs across different scales. The macroscopic design (**left**) shows a basic transistor structure with N- or P-doped nanocellulose integrated with graphene oxide nanocellulose (GO-NC) layers. At the microscopic level (**centre**), the diagram suggests replacing traditional wires with DNA strands to enhance sustainability at the molecular level in order to form gates (e.g., NAND). At the nanoscale (**right**), the image illustrates the potential for miniaturisation, showcasing a single-electron transistor (SET) configuration using nanocellulose, graphene oxide components, and a graphene back plate as a gate.

Additionally, the potential for *single-electron transistor* (SET) properties in NC was suggested by Bencurova et al. [21], indicating the material’s ability to support cutting-edge electronic components by further scaling down the technology, as shown in Figure 4. As shown in [86], the current flow can be controlled by the electric field due to the voltage applied to the gate, modulating the electron tunnelling probability from the source to the drain terminals. These studies collectively underscore the versatility and promise of NC in semiconductor applications and advancing the development of environmentally friendly and efficient electronic devices.

## 4. Electronic Design Automation for Nanocellulose-Based Technology

**Figure 5:**
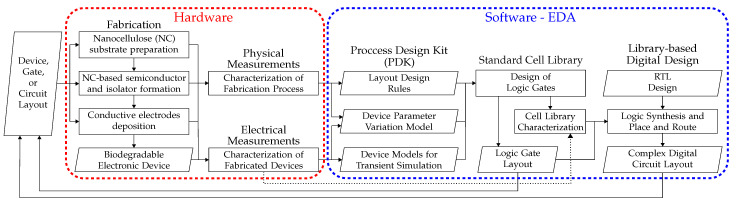
Proposed process flow for the design and fabrication of complex digital circuits using nanocellulose-based transistors. Artifacts are indicated by parallelograms, and processes are indicated by rectangles. Processes are adapted to nanocellulose composites, including transistors made from nanocellulose.

In this section, we outline the conceptual framework for the design and operation of a production line for NC compound chips. The workflow of a production line for nanocellulose transistors, logic gates, and complex digital circuits is shown in Figure 5.

Figure 5 illustrates the steps in the development of an electronic design automation solution for the fabrication of nanocellulose-based complex digital circuits such as microprocessors. Organic, biodegradable microprocessors can enable the deployment of transient wireless smart sensor systems that do not require their retrieval for correct disposal. Applications range from invasive health monitoring devices [87] to remote agricultural sensors. Kurth et al. presented a plant monitoring system using partially biodegradable wireless sensor nodes [88]. While the sensor elements are fabricated using biodegradable materials, the signal processing and wireless communication sides of the sensor nodes are built using conventional, inorganic semiconductor chips. We aim to achieve a completely biodegradable solution. In the following, we describe the foreseen workflow, as presented in Figure 5.

- **Fabrication:** The first step towards such a solution is to fabricate a single transistor using a nanocellulose-based fabrication process. The production of nanocellulose-based conductors and semiconductors is described in Figure 3.
- **Process Design Kit:** The precision of the fabrication steps must be evaluated via measurements of the physical dimensions of the transistors in comparison to the intended dimensions, described in the device layout. The limits of precision of the fabrication process are used to define the layout design rules. These rules include, for example, the minimum width and length of the electrodes, as well as the minimum spacing between two electrodes. Transistors of multiple sizes are fabricated and electrically characterised in order to construct the device models used for software simulation. The parasitic elements, namely resistors and capacitors, resulting from the wire conductors and contact capacitances of the fabricated devices are also characterised and modelled. The collection of models for the fabricated device is called a process design kit (PDK). Furthermore, the expected variation models for device parameters such as dielectric thickness and channel width are also included in the PDK. The PDK is at the most fundamental level of abstraction of digital design, namely the transistor level.
- **Standard Cell Library:** The next level of abstraction is the logic-gate level, where multiple transistors are connected to perform basic logic operations, such as NOT, AND, and OR. It is important to note that any complex logic function can be implemented using a single type of logic gate, for instance, the NOT-AND (NAND) gate. Such universal gates enable the implementation of any logic computation, although not the most efficient or fast implementation. To increase the performance of a computation, current commercial cell libraries for silicon-based chips include tens of basic logic functionalities with a large variety of sizes and threshold voltages. Chang et al. presented the first open-source cell library for organic thin-film transistors (OTFTs) The library is composed of six basic logic gates, but only p-type transistors are considered [51].
- **Cell Library-Based Digital Design:** The highest level of abstraction discussed in this survey is the module level. In this context, a module is a set of appropriately interconnected logic gates (defined in the cell library) that perform complex computations, ranging from additions to multiplications up to a complete microprocessor. Module functionality is most often described in a Register Transfer Level (RTL) hardware description language such as Verilog or VHDL. Hardware synthesis tools such as Yosys (open source) [89], or Synopsys Design Compiler (commercial) transform the RTL description into a specific sequence of logic gates and their connections. The importance of the cell library is in the reduction of the design space considered by the synthesis tool in order to reach a feasible solution in a manageable time frame. Then, so-called Place and Route (PNR) tools position the synthesized logic gates in an empty circuit layout and define the exact routing from gate to gate such that non-idealities, such as wiring delays, are minimised. The final result is a fabrication layout in Graphic Design System (GDS) format describing the exact position of each transistor (gates are made of transistors) and each wire connection such that the intended module functionality is performed by the fabricated module.

The creation of an EDA tool chain for organic, fully biodegradable circuits is driven by the need to expand application possibilities and address the growing issue of electronic waste (e-waste). Advances in organic material engineering have enabled the development of flexible, low-cost, and large-area circuits that can be printed on various substrates, like biodegradable plastics, paper, and NC, with applications in large-area sensing, artificial skin, and on-body wearables. However, challenges such as limited transistor density and performance variations due to the printing process persist. A PDK can mitigate these issues by modelling transistor behaviour and integrating these data into downstream EDA tools, facilitating complex circuit design. Moreover, biodegradable organic electronics offer an environmentally friendly alternative to traditional electronics, decomposing harmlessly and reducing e-waste. Despite the lower electron mobility and stability challenges of organic semiconductors, a comprehensive PDK including a digital standard cell library and an integrated RTL-to-GDS flow can streamline the design of large-scale organic circuits, promoting the development of sustainable electronics and integration into existing EDA flows.

## 5. Further Extensions of the Nanocellulose Composite for Improved Storage and Operation

NC is an excellent composite material with versatile applications, particularly in integrating DNA for long-term (cold) storage. Light-gated enzymes can be used to operate such DNA storage, offering an environmentally friendly long-term memory solution efficiently controlled by light [21]. However, a review of our concept of nanocellulose chip fabrication suggests the full integration of light-gated protein domains in NC chips. This extends their functionalities, including those of fluorescent proteins, energy levels (ATP), charge (pH), and other biophysical properties. Importantly, such domains also facilitate the operation of pores in NC chips for the exchange of materials, ions, charges, and information. Following the printing of the central electronics, additional biochemical capabilities can be integrated into the NC chip, which can be directly controlled by light. This light gating or emission to activate the enzymes and pores can be provided by standard LEDs or LEDs produced according the principles of green paper electronics [39], enhancing environmental control over biophysical variables and enzymes in the NC chip. In such applications, the role of the NC is as a substrate only because the moisture hydrogel can then negatively affect the folding of proteins, as well as their functional properties.

- **DNA as long-term storage:** NC preserves DNA effectively, with no degradation over 24 months [21]. However, the full NC compound improves protection further. Under suitable optimised conditions, DNA can be preserved with error codes for thousands of years [90].
  Repair enzymes and their substrates also remain stable in NC, suggesting that DNA preservation may be even further improved with active repair and maintenance, akin to natural processes [91]. NC serves as an excellent host material for these preservation efforts.
- **Specific light-gated enzymes to operate DNA as long-term storage:** The efficient use of DNA storage requires nucleotide processing enzymes for read-in and read-out. These enzymes can be rendered light-gated, controlled by domains such as BLUF, LOV, and LOV2, which respond to visible light by altering their structure activating the enzyme fused to them. An example is LOV-*Taq* polymerase, where the LOV domain controls *Taq* polymerase activity, extending the DNA strand according to a template sequence. The BLUF domain, activated by blue light, offers intrinsic OFF switching after a set time (30 min; modifiable to 5 min through protein engineering [21]). Current efforts focus on optimising these enzyme properties through protein engineering approaches (e.g., [92,93]) to maintain and operate DNA storage in NC.
- **Transparent display from nanocellulose:** Transparent NC provides the protective layer of the display, ensuring durability and functionality, as well as foldability, and using OLED for light emission [94] or using electrochromic display screens printed transparent nanocellulose-based substrates [95]. By integrating these advanced features, NC chips can achieve a high degree of functionality and sustainability, making them suitable for a wide range of applications in semiconductor technologies and beyond.

## 6. Discussion

Our survey shows that NC technology is mature enough to enable the establishment of production lines for green NC chips. In addition to conductive nanocellulose-based materials that can be made available by integrating graphene oxide [57], n- and p-type semiconducting NC composites are also available [45,50]. The addition of coatings to nanoparticles improves suspension and substrate production. The high durability of silver particles improves the composite’s flexibility, making it a more suitable, substrate even under high pressure. The smooth, even surface created by metal oxides helps to improve the adhesion of electronic components. These coatings provide electrical conductivity, mechanical flexibility, and multifunctional capabilities, making nanocellulose-based substrates ideal for use in flexible electronics, wearables, optoelectronics, and biomedical devices. The necessary material types are available to implement a full NC transistor, as illustrated in Figure 2. We also consider long-term goals, as NC shows potential for miniaturisation down to SETs [21]. Moreover, light-gated enzymes [93] may form a basis for the integration of the active operation of DNA storage in NC composite.

When implementing a production line for NC composite chips (Figure 5), the cutting-edge use of conductive patterns [96] is important. To this end, we here, we collect the latest published data from different laboratories (Figure 1). Table 1 and Table 2 summarise how this is supported by recent progress by other groups with respect to sustainable microelectronics devices and the use of nanocellulose-based materials for organic transistors, as well as the derived EDA tool chains.

In Table 3, we summarise various advantages and disadvantages of nanocellulose composites for a novel, environmentally friendly, chip design with an easy-to-use automated production process. Despite the promising properties of NC, such as renewability and mechanical strength, integrating NC into chip technology poses multiple challenges. The most significant issue is its high water retention and sensitivity due to its hydrophilic nature. While this property is beneficial in applications like hydrogels for cosmetics and food, it is highly detrimental for use in electronics, where moisture can cause swelling, alter the chip’s properties, and even lead to short circuits. Reducing NC’s hydrophilicity through chemical treatments could be a crucial step in making it viable for sustainable electronics.

On the other hand, NC can be sourced from sustainable materials such as microorganisms, agricultural waste, and recycled textiles, making it an attractive option for green manufacturing. However, some processing methods require large amounts of water and harmful chemicals, leading to environmental concerns and high energy consumption. Additionally, thermal degradation of NC fibres is a challenge, as they are stable up to 250 °C but degrade at higher temperatures, which are common in chip production, thereby limiting processing options [97]. This can negatively influence the manufacturing process, including the integration of NC with other materials that are required for chip production. In typical microchip production, the temperature can reach up to 1000 °C, and this thermal limitation restricts the choice of processing techniques.

While NC offers numerous advantages (see Table 3) for sustainable and innovative chip design, such challenges must be addressed to optimize its potential. NC’s natural tendency to form hydrogels, absorbing up to 250 g of water per gram of NC, poses a challenge in maintaining consistent mechanical properties and low moisture levels in electronic applications [98]. The significant challenge here remains to maintain consistent mechanical properties and low moisture levels without forming agglomerations. Irreversible agglomerates can be formed after rehydration due to strong hydrogen bonding between the nanocellulose fibres. Dewatering of the NC is a challenging process, although several methods have already been developed. Common drying methods include oven drying, spray drying, freeze drying, and supercritical CO_2_ drying, as well as their combinations, like spray freeze drying and supercritical CO_2_ spray drying. Filtration is one of the most common methods, but nanocellulose tends to clump together due to strong hydrogen binding. In this method, NC passes through a porous medium using vacuum or pressure; however, the fibre sizes are often uneven, and the procedure is slow, especially for high-viscosity suspensions [99,100]. Oven drying is relatively fast and cost-effective; however, it can change the structure of the nanocellulose and lead to fibre aggregation and inconsistent surface properties [101]. Drying techniques such as spray drying and freeze drying are more suitable for the production of stable NC; however, scalability and particle aggregation are also problematic here [101,102,103]. On the other hand, supercritical CO_2_ drying keeps the fine NC structure after drying, which is desired, mainly for the production of aerogels. In this method, water is replaced with ethanol, which has a more than 10 times lower critical point than that of water. Therefore, the critical point for CO_2_ does not influence NC fibres, and ethanol can be easily displaced by CO_2_ at ambient temperature under high pressure. However, this method can also change the mechanical properties of the NC, such as the size of the nanofibres [103]. To prevent aggregation, various chemical agents are used, like xanthan, sucrose, and carboxymethyl cellulose. For example, the use of tert-butanol as a co-solvent reduces drying times and maintains NC dispersibility [104,105], while polyvinyl alcohol acts as a capping agent, enhancing the specific surface area of oven-dried NC and preventing agglomeration [106]. Although these chemical treatments can be effective, they may have limitations for large-scale applications and could require additional processing steps to remove the additives, potentially affecting the final properties of the NC.

Inspired by recent progress regarding carbon nanotube chips, which hold promise for more energy-efficient computing systems in the future, we advocate for an era of nanocellulose-based chip design.

The key to achieving further progress beyond previous efforts and publications is to produce a transistor completely made from NC. Establishing such a transistor opens up all the attractive possibilities of current automated chip design for silicon wafer technology, such as full scalability and downsizing and the efficient and smooth production of even more transistors on one wafer.

Hence, here, we show that a transistor made exclusively from NC is within reach, as shown in Figure 2. While pure NC has already been shown to be capable of functioning as an appropriate substrate and dielectric layers, we go further to propose that all transistor layers (substrate, dielectric, conductor, and semiconductors) can be directly derived from NC (see Table 1 and Figure 3).

Carrying this innovative main concept of a fully NC-based transistor further, we next show how the fully automated production of such a full NC chip would look, including a process design kit (PDK), expected variation models, and a standard cell library (Figure 5). We are aware that, as with previous efforts with respect to other types of chip design away from classical silicon wafers, such as carbon nanotubes ([107] or [108]), the development of such a full nanocellulose chip will take a couple of years, starting from the optimisation of the nanocellulose-based transistor to parameterisation of optimal chip-design steps. However, we show here that the potential is there; all steps and components have solid experimental data behind them to show that they can be assembled to implement transistors.

## 7. Outlook and Conclusions

Using these steps as a basis, the outlook includes (i) the use of fully conducting NC composites with graphene for all conductor tracks, (ii) the use of nanocellulose chips to control automated pipelines and nanofactories, (iii) the integration of light-gated enzymes in NC chips for efficient operation of long-term memory in the form of DNA storage, and (iv) the integration of light-gated ion pores in NC compounds to control the biochemical environment (e.g., pH), achieving battery and energy generation and allowing for even more sophisticated reactions controlled by NC chips. These future applications each need independent development but can be explored and directly tackled as soon an efficiently produced NC chip is available.

In summary, we make the following conclusions:

(i) In this survey, we argue for the feasibility of constructing a high-performance nanocellulose-based, using nanocellulose for the dielectric, conductive, and semiconductive layers. Our findings indicate that p-type and n-type nanocellulose materials are already available, suggesting that the development of an entirely cellulose-based transistor is imminent. Such a development would be a game changer, as it promises a fully biodegradable alternative to conventional electronics, eliminating the use of toxic inks and other materials.
(ii) Nanocellulose’s properties extend beyond semiconductivity. This versatility allows for the integration of DNA for long-term or cold storage, making use of light-gated enzymes for DNA storage. This review highlights the significant potential for further improvement for instance, new light-gated enzyme constructs and modifications of fluorescence by different fluorescent proteins.
(iii) Additionally, the potential for miniaturisation of fully nanocellulose transistors towards single-electron transistors (SETs) represents a crucial advancement in this technology.
(iv) Moreover, the design automation steps outlined in this paper, when considered alongside the current literature on material science and chip fabrication, provide a clear pathway for the advancement of nanocellulose-based electronics. This comprehensive approach not only emphasises the readiness of the necessary components but also paves the way for future innovations in environmentally friendly and sustainable electronic devices.

## Figures and Tables

**Table 2:** Summary of recent research achievement with respect to organic and cellulose-based transistors and circuits, as well as their associated EDA tools and required resources for integrated circuit design and fabrication.

Recent Research Regarding Organic and Cellulose-Based Transistors and Circuits						
Year	Reference	Transistor	Inverter	Logic Gates	PDK	Cell Library
2024	[39]	P & N-OFET	Yes	Yes		
	[40]	P-OFET	Yes	Yes	Yes	Yes
2023	[54]	P-OFET	Yes	Yes		
	[41]	P-OFET				
	[42]	P-OFET				
	[43]	N-OFET				
2022	[48]	N-OFET				
	[47]	P-OFET	Yes			
2021	[49]	N-OFET				
	[55]	P- & N-OFET	Yes			
2020	[51]	P-OFET	Yes	Yes	Yes	Yes
2019	[56]	P- & N-OFET	Yes	Yes		
2018	[52]	P- & N-OFET	Yes			
2016	[53]	P-OFET				
2014	[37]	P & N-OFET				

**Table 3:** Advantages and challenges of nanocellulose chip design and production.

Advantages		Disadvantages	
Advantage	Comment	Problem	Possible Solutions
Ideal host and composite material	Can be easily obtained from sustainable material (bacteria, food, organic agricultural waste, wood, plants, etc.)	Challenging to obtain an exact shape	Cutting with LASER and 3D printing, with bacterial nanocellulose growing in the moulds
Easy to manufacture for special features	Conductivity, optical transparency, stiffness, and flexibility	Fibre stability and thermal degradation at high temperatures	Application only in mid-temperature devices (under 250 °C)
Broad applications in chip technology	Various phases of the final product (aerogel, gel, never-dried membrane, solid membrane, and hard material)	Low conductivity	Treatment with iodine, graphene, or nanometal particles
Various design possibilities	E.g., origami and kirigami design [46]	High price (processing and fabrication)	Automation of the manufacturing process
Scalability	Material can be easily scaled-up in an environmentally sustainable way	Biodegradability can be too high	Treatment of the nanocellulose with anti-microbial components
Biodegradability	Easy to degrade and compost and very environmentally friendly	Compatibility with other materials	Due to the hydrophilic nature of nanocellulose, it is incompatible with hydrophobic materials such as petroleum-based products. The proper polymer has to be used or nanocellulose has to be treated to alter the hydrophilic surface prior to linking with hydrophobic material.

## Data Availability

All results are contained in the manuscript including all details on the involved modelling and all experimental data generated in the study.

## Abbreviations

The following abbreviations are used in this manuscript:

BNC	Bacterial nanocellulose
DNA	Deoxynucleic acid
EDA	Electronic design automation
FET	Field-effect transistor
GDS	Graphic design system
GO	Graphene oxide
ICCN	Ionic conductive cellulose nanopaper
LGENC	Low-oxidized graphene/nanocellulose hybrid
NC	Nanocellulose
OFET	Organic field-effect transistor
OTFT	Organic thin-film transistor
PANI	Polyaniline
PDK	Process design kit
PNR	Place and route
PPy	Polypyrrole
RTL	Register transfer level
SET	Single-electron transistor
TFT	Thin-film transistor

## References

[1] Kondo T. (2005). Hydrogen bonds in cellulose and cellulose derivatives. Polysaccharides: Structural Diversity and Functional Versatility.

[2] Klemm D., Heublein B., Fink H.P., Bohn A. (2005). Cellulose: Fascinating biopolymer and sustainable raw material. Angew. Chem. Int. Ed..

[3] Ghilan A., Nicu R., Ciolacu D.E., Ciolacu F. (2023). Insight into the Latest Medical Applications of Nanocellulose. Materials.

[4] Qi Y., Guo Y., Liza A.A., Yang G., Sipponen M.H., Guo J., Li H. (2023). Nanocellulose: A review on preparation routes and applications in functional materials. Cellulose.

[5] Samyn P., Meftahi A., Geravand S.A., Heravi M.E.M., Najarzadeh H., Sabery M.S.K., Barhoum A. (2023). Opportunities for bacterial nanocellulose in biomedical applications: Review on biosynthesis, modification and challenges. Int. J. Biol. Macromol..

[6] Poletto M., Ornaghi H.L., Zattera A.J. (2014). Native Cellulose: Structure, Characterization and Thermal Properties. Materials.

[7] Marín P., Martirani-Von Abercron S.M., Urbina L., Pacheco-Sánchez D., Castañeda-Cataña M.A., Retegi A., Eceiza A., Marqués S. (2019). Bacterial nanocellulose production from naphthalene. Microb. Biotechnol..

[8] Abba M., Nyakuma B.B., Ibrahim Z., Ali J.B., Razak S.I.A., Salihu R. (2022). Physicochemical, morphological, and microstructural characterisation of bacterial nanocellulose from Gluconacetobacter xylinus BCZM. J. Nat. Fibers.

[9] Tanskul S., Amornthatree K., Jaturonlak N. (2013). A new cellulose-producing bacterium, *Rhodococcus* sp. MI 2: Screening and optimization of culture conditions. Carbohydr. Polym..

[10] Castiblanco L.F., Sundin G.W. (2018). Cellulose production, activated by cyclic di-GMP through BcsA and BcsZ, is a virulence factor and an essential determinant of the three-dimensional architectures of biofilms formed by Erwinia amylovora Ea1189. Mol. Plant Pathol..

[11] da Gama F.M.P., Dourado F. (2018). Bacterial NanoCellulose: What future?. BioImpacts BI.

[12] Fernandez Corujo V.L., Arroyo S.M., Cerrutti P., Foresti M.L. (2023). Production of bacterial nanocellulose in alternative culture media under static and dynamic conditions. Lat. Am. Appl. Res..

[13] Iguchi M., Yamanaka S., Budhiono A. (2000). Bacterial cellulose—A masterpiece of nature’s arts. J. Mater. Sci..

[14] Sardjono S.A., Suryanto H., Aminnudin A., Muhajir M. (2019). Crystallinity and morphology of the bacterial nanocellulose membrane extracted from pineapple peel waste using high-pressure homogenizer. AIP Conf. Proc..

[15] Mohite B.V., Patil S.V. (2014). Physical, structural, mechanical and thermal characterization of bacterial cellulose by G. hansenii NCIM 2529. Carbohydr. Polym..

[16] Rana A.K., Thakur V.K. (2023). Impact of physico-chemical properties of nanocellulose on rheology of aqueous suspensions and its utility in multiple fields: A review. J. Vinyl Addit. Technol..

[17] Jing S., Wu L., Siciliano A.P., Chen C., Li T., Hu L. (2023). The critical roles of water in the processing, structure, and properties of nanocellulose. ACS Nano.

[18] Börjesson M., Westman G. (2015). Crystalline nanocellulose—Preparation, modification, and properties. Cellulose—Fundamental Aspects and Current Trends.

[19] Das J., Mishra H.N. (2023). Electrochemical biosensor for monitoring fish spoilage based on nanocellulose as enzyme immobilization matrix. J. Food Meas. Charact..

[20] Malarat S., Khongpun D., Limtong K., Sinthuwong N., Soontornapaluk P., Sakdaronnarong C., Posoknistakul P. (2023). Preparation of nanocellulose from coffee pulp and its potential as a polymer reinforcement. ACS Omega.

[21] Bencurova E., Shityakov S., Schaack D., Kaltdorf M., Sarukhanyan E., Hilgarth A., Rath C., Montenegro S., Roth G., Lopez D. (2022). Nanocellulose composites as smart devices with chassis, light-directed DNA storage, engineered electronic properties, and chip integration. Front. Bioeng. Biotechnol..

[22] Xiao J., Wu F., Hu C., Zhu Z., Liu B. (2024). Nanocellulose aerogel prepared using discarded cotton textiles: For constructing water evaporation and self-cooling thermoelectric systems. Ind. Crop. Prod..

[23] Cai Y., Yang Q., Wang E., Liang Y., Han W., Miao Y., Huang J., Zhang W. (2024). Developing cellulose nanofibrils/Na-montmorillonite composite air filter with efficient filtration ability for PM2. 5 and adsorption of formaldehyde. Appl. Surf. Sci..

[24] Shang M., Yu J., Li J., Huang P., Jia X., Miao W., Tian L., Sun H., Dai Y., Zhang L. (2024). Cellulose/Poly (Vinyl Alcohol)/Graphene Composite Photothermal Aerogel Membrane for Solar-driven Seawater Desalination. Waste Biomass Valorization.

[25] Jung Y.H., Chang T.H., Zhang H., Yao C., Zheng Q., Yang V.W., Mi H., Kim M., Cho S.J., Park D.W. (2015). High-performance green flexible electronics based on biodegradable cellulose nanofibril paper. Nat. Commun..

[26] Fang Z., Zhang H., Qiu S., Kuang Y., Zhou J., Lan Y., Sun C., Li G., Gong S., Ma Z. (2021). Versatile Wood Cellulose for Biodegradable Electronics. Adv. Mater. Technol..

[27] Yang H., Zheng H., Duan Y., Xu T., Xie H., Du H., Si C. (2023). Nanocellulose-graphene composites: Preparation and applications in flexible electronics. Int. J. Biol. Macromol..

[28] Ali A.S., Zaidan K.M., Al-Badran A.I. (2022). Preparation of Poly (methyl methacrylate) thin film Capacitors on ITO-glass substrate. MJPS.

[29] Pak K., Seong H., Choi J., Hwang W.S., Im S.G. (2016). Synthesis of ultrathin, homogeneous copolymer dielectrics to control the threshold voltage of organic thin-film transistors. Adv. Funct. Mater..

[30] Konwar G., Saxena P., Raghuwanshi V., Rahi S., Tiwari S.P. (2022). Multifunctional Flexible Organic Transistors with a High-k/Natural Protein Bilayer Gate Dielectric for Circuit and Sensing Applications. ACS Appl. Electron. Mater..

[31] Xie Y., Ding C., Jin Q., Zheng L., Xu Y., Xiao H., Cheng M., Zhang Y., Yang G., Li M. (2024). Organic transistor-based integrated circuits for future smart life. SmartMat.

[32] Zschieschang U., Klauk H. (2019). Organic transistors on paper: A brief review. J. Mater. Chem. C.

[33] Jia D., Xie J., Dirican M., Fang D., Yan C., Liu Y., Li C., Cui M., Liu H., Chen G. (2022). Highly smooth, robust, degradable and cost-effective modified lignin-nanocellulose green composite substrates for flexible and green electronics. Compos. Part B Eng..

[34] Huang J., Zhu H., Chen Y., Preston C., Rohrbach K., Cumings J., Hu L. (2013). Highly transparent and flexible nanopaper transistors. ACS Nano.

[35] Sabo R., Seo J.H., Ma Z. (2012). Cellulose nanofiber composite substrates for flexible electronics. Proceedings of the 2012 TAPPI International Conference on Nanotechnology for Renewable Materials.

[36] Seo J.H., Chang T.H., Lee J., Sabo R., Zhou W., Cai Z., Gong S., Ma Z. (2015). Microwave flexible transistors on cellulose nanofibrillated fiber substrates. Appl. Phys. Lett..

[37] Gaspar D., Fernandes S.N., de Oliveira A.G., Fernandes J.G., Grey P., Pontes R.V., Pereira L., Martins R., Godinho M.H., Fortunato E. (2014). Nanocrystalline cellulose applied simultaneously as the gate dielectric and the substrate in flexible field effect transistors. Nanotechnology.

[38] Betker M., Erichlandwehr T., Sochor B., Erbes E., Kurmanbay A., Alon Y., Li Y., Fernandez-Cuesta I., Müller-Buschbaum P., Techert S.A. (2024). Micrometer-Thin Nanocellulose Foils for 3D Organic Electronics. Adv. Funct. Mater..

[39] Rafiee Z., Elhadad A., Choi S. (2024). Revolutionizing Papertronics: Advanced Green, Tunable, and Flexible Components and Circuits. Adv. Sustain. Syst..

[40] Gupta P., Lukosiunas J., Marques G.C., Raths S., Stehlin S., Schlisske S., Exner K., Strunk K.P., Melzer C., Erk P. (2024). Active matrix-based pressure sensor system with a 4 × 16 printed decoder designed with a flexible hybrid organic process design kit. Flex. Print. Electron..

[41] Konwar G., Rahi S., Tiwari S.P. (2023). Decomposable Flexible Organic Transistors with a Cellulose-Based Gate Dielectric and Substrate for Biodegradable Electronics. ACS Appl. Mater. Interfaces.

[42] Konwar G., Rahi S., Tiwari S.P. (2023). Exploration of a Cellulose-Based Biocompatible Gate Dielectric for Low-Voltage Organic Transistors. IEEE J. Flex. Electron..

[43] Lu S., Smith B.N., Meikle H., Therien M.J., Franklin A.D. (2023). All-Carbon Thin-Film Transistors Using Water-Only Printing. Nano Lett..

[44] Thi Q.V., Ko J., Jo Y., Joo Y. (2022). Ion-Incorporative, Degradable Nanocellulose Crystal Substrate for Sustainable Carbon-Based Electronics. ACS Appl. Mater. Interfaces.

[45] Vasheghani Farahani M.S., Nikzad M., Ghorbani M. (2022). Fabrication of Fe-doped ZnO/nanocellulose nanocomposite as an efficient photocatalyst for degradation of methylene blue under visible light. Cellulose.

[46] Koga H., Nagashima K., Suematsu K., Takahashi T., Zhu L., Fukushima D., Huang Y., Nakagawa R., Liu J., Uetani K. (2022). Nanocellulose Paper Semiconductor with a 3D Network Structure and Its Nano–Micro–Macro Trans-Scale Design. ACS Nano.

[47] Granelli R., Alessandri I., Gkoupidenis P., Vassalini I., Kovács-Vajna Z.M., Blom P.W.M., Torricelli F. (2022). High-Performance Bioelectronic Circuits Integrated on Biodegradable and Compostable Substrates with Fully Printed Mask-Less Organic Electrochemical Transistors. Small.

[48] Dahiya A.S., Zumeit A., Christou A., Dahiya R. (2022). High-Performance n-Channel Printed Transistors on Biodegradable Substrate for Transient Electronics. Adv. Electron. Mater..

[49] Williams N.X., Bullard G., Brooke N., Therien M.J., Franklin A.D. (2021). Printable and recyclable carbon electronics using crystalline nanocellulose dielectrics. Nat. Electron..

[50] Yu J., Zhu Z., Zhang H., Qiu Y., Yin D., Cheng Y., Wang S. (2021). Stepwise carbonization of nanocellulose to N-doped carbons with structural transformation and enhanced peroxymonosulfate activation. Chem. Eng. J..

[51] Chang T.J., Yao Z., Rand B.P., Wentzlaff D. Organic-Flow: An Open-Source Organic Standard Cell Library and Process Development Kit. Proceedings of the 2020 Design, Automation & Test in Europe Conference & Exhibition (DATE).

[52] Dai S., Chu Y., Liu D., Cao F., Wu X., Zhou J., Zhou B., Chen Y., Huang J. (2018). Intrinsically ionic conductive cellulose nanopapers applied as all solid dielectrics for low voltage organic transistors. Nat. Commun..

[53] Hassinen T., Alastalo A., Eiroma K., Tenhunen T.M., Kunnari V., Kaljunen T., Forsström U., Tammelin T. (2016). All-Printed Transistors on Nano Cellulose Substrate. MRS Adv..

[54] Haldar T., Wollandt T., Weis J., Zschieschang U., Klauk H., Weitz R.T., Burghartz J.N., Geiger M. (2023). High-gain, low-voltage unipolar logic circuits based on nanoscale flexible organic thin-film transistors with small signal delays. Sci. Adv..

[55] Sharova A.S., Caironi M. (2021). Sweet Electronics: Honey-Gated Complementary Organic Transistors and Circuits Operating in Air. Adv. Mater..

[56] Kwon J., Takeda Y., Shiwaku R., Tokito S., Cho K., Jung S. (2019). Three-dimensional monolithic integration in flexible printed organic transistors. Nat. Commun..

[57] Wang R., Ma Q., Zhang H., Ma Z., Yang R., Zhu J.Y. (2019). Producing Conductive Graphene–Nanocellulose Paper in One-pot. J. Polym. Environ..

[58] Liu K., Hu J., Kong Z., Hu J., Tian Z., Hou J., Qin J., Liu C., Liang S., Wu H. (2021). High-yield, high-conductive graphene/nanocellulose hybrids prepared by Co-exfoliation of low-oxidized expanded graphite and microfibrillated cellulose. Compos. Part B Eng..

[59] Sheng N., Chen S., Zhang M., Wu Z., Liang Q., Ji P., Wang H. (2021). TEMPO-Oxidized Bacterial Cellulose Nanofibers/Graphene Oxide Fibers for Osmotic Energy Conversion. ACS Appl. Mater. Interfaces.

[60] Kim Y., Kim Y.T., Wang X., Min B., Park S.I. (2023). TEMPO-Oxidized Cellulose Nanofibril Films Incorporating Graphene Oxide Nanofillers. Polymers.

[61] Verma Y.K., Singh A.K., Paswan M., Gurmaita P.K. (2024). Preparation and characterization of bamboo based nanocellulose by ball milling and used as a filler for preparation of nanocomposite. Polymer.

[62] Mahmud M.M., Perveen A., Jahan R.A., Matin M.A., Wong S.Y., Li X., Arafat M.T. (2019). Preparation of different polymorphs of cellulose from different acid hydrolysis medium. Int. J. Biol. Macromol..

[63] Yang T., Li X., Guo Y., Zhao J., Qu Y. (2023). Preparation of nanocellulose crystal from bleached pulp with an engineering cellulase and co-production of ethanol. Carbohydr. Polym..

[64] Rana A.K., Scarpa F., Thakur V.K. (2022). Cellulose/polyaniline hybrid nanocomposites: Design, fabrication, and emerging multidimensional applications. Ind. Crop. Prod..

[65] Muller D., Silva J., Rambo C., Barra G., Dourado F., Gama F. (2013). Neuronal cells’ behavior on polypyrrole coated bacterial nanocellulose three-dimensional (3D) scaffolds. J. Biomater. Sci. Polym. Ed..

[66] Nguyen H.L., Jo Y.K., Cha M., Cha Y.J., Yoon D.K., Sanandiya N.D., Prajatelistia E., Oh D.X., Hwang D.S. (2016). Mussel-Inspired Anisotropic Nanocellulose and Silver Nanoparticle Composite with Improved Mechanical Properties, Electrical Conductivity and Antibacterial Activity. Polymers.

[67] Zhang X., Lin Z., Chen B., Zhang W., Sharma S., Gu W., Deng Y. (2014). Solid-state flexible polyaniline/silver cellulose nanofibrils aerogel supercapacitors. J. Power Sources.

[68] Theivasanthi T., Anne Christma F., Toyin A.J., Gopinath S.C., Ravichandran R. (2018). Synthesis and characterization of cotton fiber-based nanocellulose. Int. J. Biol. Macromol..

[69] Munawar R.F., Saad A.F., Othman I.S., Abid M.A.A.M., Samat K.F. (2024). Characterization of Nanocellulose from Orange Peel Waste. J. Adv. Res. Appl. Mech..

[70] Yang Y., Luo C.L., Chen X.D., Wang M. (2023). Sustainable electromagnetic shielding graphene/nanocellulose thin films with excellent joule heating and mechanical properties via in-situ mechanical exfoliation and crosslinking with cations. Compos. Sci. Technol..

[71] Dias O.A.T., Konar S., Leão A.L., Sain M. (2019). Flexible electrically conductive films based on nanofibrillated cellulose and polythiophene prepared via oxidative polymerization. Carbohydr. Polym..

[72] Kim H.J., Kwon H.J., Jeon S., Park J.W., Sunthornvarabhas J., Sriroth K. (2015). Electrical and Optical Properties of Nanocellulose Films and Its Nanocomposites. Handbook of Polymer Nanocomposites. Processing, Performance and Application: Volume C: Polymer Nanocomposites of Cellulose Nanoparticles.

[73] Hsu H.H., Zhong W. (2019). Nanocellulose-Based Conductive Membranes for Free-Standing Supercapacitors: A Review. Membranes.

[74] Liu K., Liu W., Li W., Duan Y., Zhou K., Zhang S., Ni S., Xu T., Du H., Si C. (2022). Strong and highly conductive cellulose nanofibril/silver nanowires nanopaper for high performance electromagnetic interference shielding. Adv. Compos. Hybrid Mater..

[75] Hsieh M.C., Kim C., Nogi M., Suganuma K. (2013). Electrically conductive lines on cellulose nanopaper for flexible electrical devices. Nanoscale.

[76] Pras O., Beneventi D., Chaussy D., Piette P., Tapin-Lingua S. (2013). Use of microfibrillated cellulose and dendritic copper for the elaboration of conductive films from water- and ethanol-based dispersions. J. Mater. Sci..

[77] Hu L., Zheng G., Yao J., Liu N., Weil B., Eskilsson M., Karabulut E., Ruan Z., Fan S., Bloking J.T. (2013). Transparent and conductive paper from nanocellulose fibers. Energy Environ. Sci..

[78] Kuzmenko V., Saleem A.M., Bhaskar A., Staaf H., Desmaris V., Enoksson P. (2015). Hierarchical cellulose-derived carbon nanocomposites for electrostatic energy storage. J. Phys. Conf. Ser..

[79] Wu X., Lu C., Zhang X., Zhou Z. (2015). Conductive natural rubber/carbon black nanocomposites via cellulose nanowhisker templated assembly: Tailored hierarchical structure leading to synergistic property enhancements. J. Mater. Chem. A.

[80] Yang W., Zhang Y., Liu T., Huang R., Chai S., Chen F., Fu Q. (2017). Completely Green Approach for the Preparation of Strong and Highly Conductive Graphene Composite Film by Using Nanocellulose as Dispersing Agent and Mechanical Compression. ACS Sustain. Chem. Eng..

[81] Li L., Li M., Zhang Z., Qin Y., Shui X., Xia J., Xiong S., Wang B., Zhang Z., Wei X. (2022). Robust composite film with high thermal conductivity and excellent mechanical properties by constructing a long-range ordered sandwich structure. J. Mater. Chem. A.

[82] González-Gil R.M., Borràs M., Chbani A., Abitbol T., Fall A., Aulin C., Aucher C., Martínez-Crespiera S. (2022). Sustainable and Printable Nanocellulose-Based Ionogels as Gel Polymer Electrolytes for Supercapacitors. Nanomaterials.

[83] Kowalczuk J., Bielejewski M., Tritt-Goc J. (2023). Ionic liquid dynamics and electrical conductivity under confinement within micro and nanocellulose ionogels. Cellulose.

[84] Nguyen D.T.A., Wang L., Imae T., Su C.J., Jeng U.S., Rojas O.J. (2024). Nanoarchitectonics of Nanocellulose Filament Electrodes by Femtosecond Pulse Laser Deposition of ZnO and In Situ Conjugation of Conductive Polymers. ACS Appl. Mater. Interfaces.

[85] Gutierrez J., Tercjak A., Algar I., Retegi A., Mondragon I. (2012). Conductive properties of TiO_2_/bacterial cellulose hybrid fibres. J. Colloid Interface Sci..

[86] Shityakov S., Roewer N., Förster C., Broscheit J.A. (2017). In Silico Modeling of Indigo and Tyrian Purple Single-Electron Nano-Transistors Using Density Functional Theory Approach. Nanoscale Res. Lett..

[87] Surya S.G., Raval H.N., Ahmad R., Sonar P., Salama K.N., Rao V. (2019). Organic field effect transistors (OFETs) in environmental sensing and health monitoring: A review. TrAC Trends Anal. Chem..

[88] Kurth S., Voigt S., Zichner R., Roscher F., Weigel P., Großmann T. Technologies for biodegradable wireless plant monitoring sensors. Proceedings of the 2021 Smart Systems Integration (SSI).

[89] Wolf C., Glaser J., Kepler J. Yosys-a free Verilog synthesis suite. Proceedings of the 21st Austrian Workshop on Microelectronics (Austrochip).

[90] Grass R.N., Heckel R., Puddu M., Paunescu D., Stark W.J. (2015). Robust Chemical Preservation of Digital Information on DNA in Silica with Error-Correcting Codes. Angew. Chem. Int. Ed..

[91] Islam S., Peart C., Kehlmaier C., Sun Y.H., Lei F., Dahl A., Klemroth S., Alexopoulou D., del Mar Delgado M., Laiolo P. (2024). Museomics help resolving the phylogeny of snowfinches (Aves, Passeridae, Montifringilla and allies). Mol. Phylogenetics Evol..

[92] Shen H., Lynch E.M., Akkineni S., Watson J.L., Decarreau J., Bethel N.P., Benna I., Sheffler W., Farrell D., DiMaio F. (2024). De novo design of pH-responsive self-assembling helical protein filaments. Nat. Nanotechnol..

[93] Salihoglu R., Srivastava M., Liang C., Schilling K., Szalay A., Bencurova E., Dandekar T. (2023). PRO-Simat: Protein network simulation and design tool. Comput. Struct. Biotechnol. J..

[94] Xie J., Jia D., Dirican M., Xia Y., Li C., Liu Y., Cui M., Yan C., Wan J., Liu H. (2022). Highly Foldable, Super-Sensitive, and Transparent Nanocellulose/Ceramic/Polymer Cover Windows for Flexible OLED Displays. ACS Appl. Mater. Interfaces.

[95] Andersson Ersman P., Freitag K., Nilsson M., Åhlin J., Brooke R., Nordgren N., Aulin C., Fall A., Nevo Y., Beni V. (2023). Electrochromic Displays Screen Printed on Transparent Nanocellulose-Based Substrates. Adv. Photonics Res..

[96] Ko Y., Kwon G., Choi H., Lee K., Jeon Y., Lee S., Kim J., You J. (2023). Cutting Edge Use of Conductive Patterns in Nanocellulose-Based Green Electronics. Adv. Funct. Mater..

[97] Lichtenstein K., Lavoine N. (2017). Toward a deeper understanding of the thermal degradation mechanism of nanocellulose. Polym. Degrad. Stab..

[98] Barajas-Ledesma R.M., Patti A.F., Wong V.N., Raghuwanshi V.S., Garnier G. (2020). Engineering nanocellulose superabsorbent structure by controlling the drying rate. Colloids Surf. A Physicochem. Eng. Asp..

[99] Lindström T. (2021). A Proposition for the Estimation of the Maximum Tensile Strength of Variously Charged Nanocellulosic Film Materials Provided by Vacuum Filtration. Nanomaterials.

[100] Reid M.S., Suganda W., Östmark E., Brolin A., Wågberg L. (2023). Dewatering of Micro- and Nanofibrillated Cellulose for Membrane Production. ACS Sustain. Chem. Eng..

[101] Beck S., Bouchard J., Berry R. (2012). Dispersibility in water of dried nanocrystalline cellulose. Biomacromolecules.

[102] Esparza Y., Ngo T.D., Fraschini C., Boluk Y. (2019). Aggregate morphology and aqueous dispersibility of spray-dried powders of cellulose nanocrystals. Ind. Eng. Chem. Res..

[103] Peng Y., Gardner D.J., Han Y. (2012). Drying cellulose nanofibrils: In search of a suitable method. Cellulose.

[104] Hanif Z., Jeon H., Tran T.H., Jegal J., Park S.A., Kim S.M., Park J., Hwang S.Y., Oh D.X. (2018). Butanol-mediated oven-drying of nanocellulose with enhanced dehydration rate and aqueous re-dispersion. J. Polym. Res..

[105] Jiang F., Hsieh Y.L. (2014). Assembling and redispersibility of rice straw nanocellulose: Effect of tert-butanol. ACS Appl. Mater. Interfaces.

[106] Velásquez-Cock J., Posada P., Castro C., Gañán P., Zuluaga R., Gómez H.B.E., Serpa G.A., Gómez H.C. (2018). Poly (vinyl alcohol) as a capping agent in oven dried cellulose nanofibrils. Carbohydr. Polym..

[107] Shulaker M.M., Hills G., Patil N., Wei H., Chen H.Y., Wong H.S.P., Mitra S. (2013). Carbon nanotube computer. Nature.

[108] Xia F., Xia T., Xiang L., Ding S., Li S., Yin Y., Xi M., Jin C., Liang X., Hu Y. (2022). Carbon Nanotube-Based Flexible Ferroelectric Synaptic Transistors for Neuromorphic Computing. ACS Appl. Mater. Interfaces.

